# Supplementary figures and images for: Supraclavicular brown adipocytes originate from Tbx1+ myoprogenitors
Source: PLoS Biol. 2023 Dec 4;21(12):e3002413. doi: 10.1371/journal.pbio.3002413 (PMC10721186; doi:10.1371/journal.pbio.3002413)

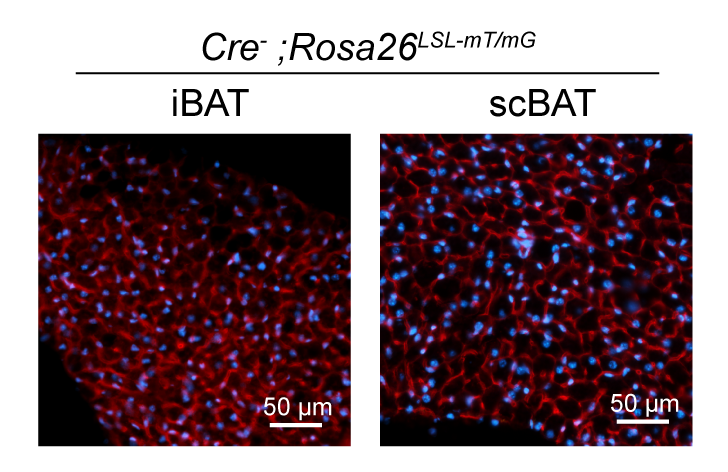

Supplement: S1 Fig — Representative fluorescent images of interscapular (left) and supraclavicular (right) BAT from Cre-negative mTmG reporter mice (scale = 50 μm). (TIF) [file pbio.3002413.s001.tif]

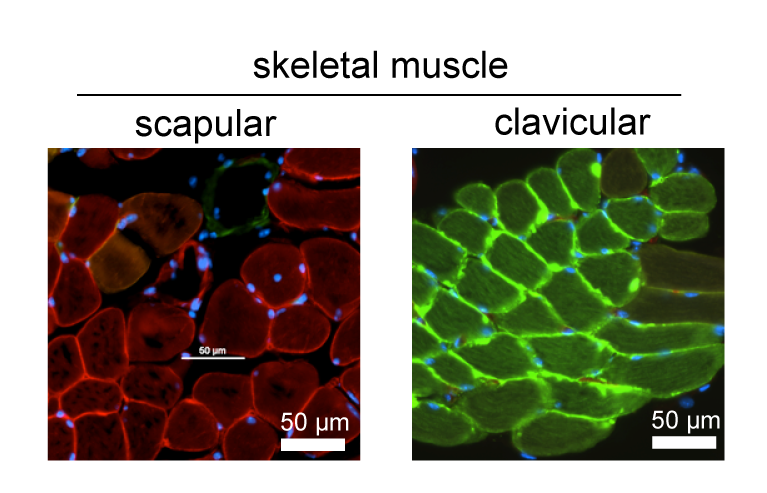

Supplement: S2 Fig — Representative fluorescent images of scapular (left) and clavicular (right) skeletal muscles from Tbx1-mTmG reporter mice (scale = 50 μm). (TIF) [file pbio.3002413.s002.tif]

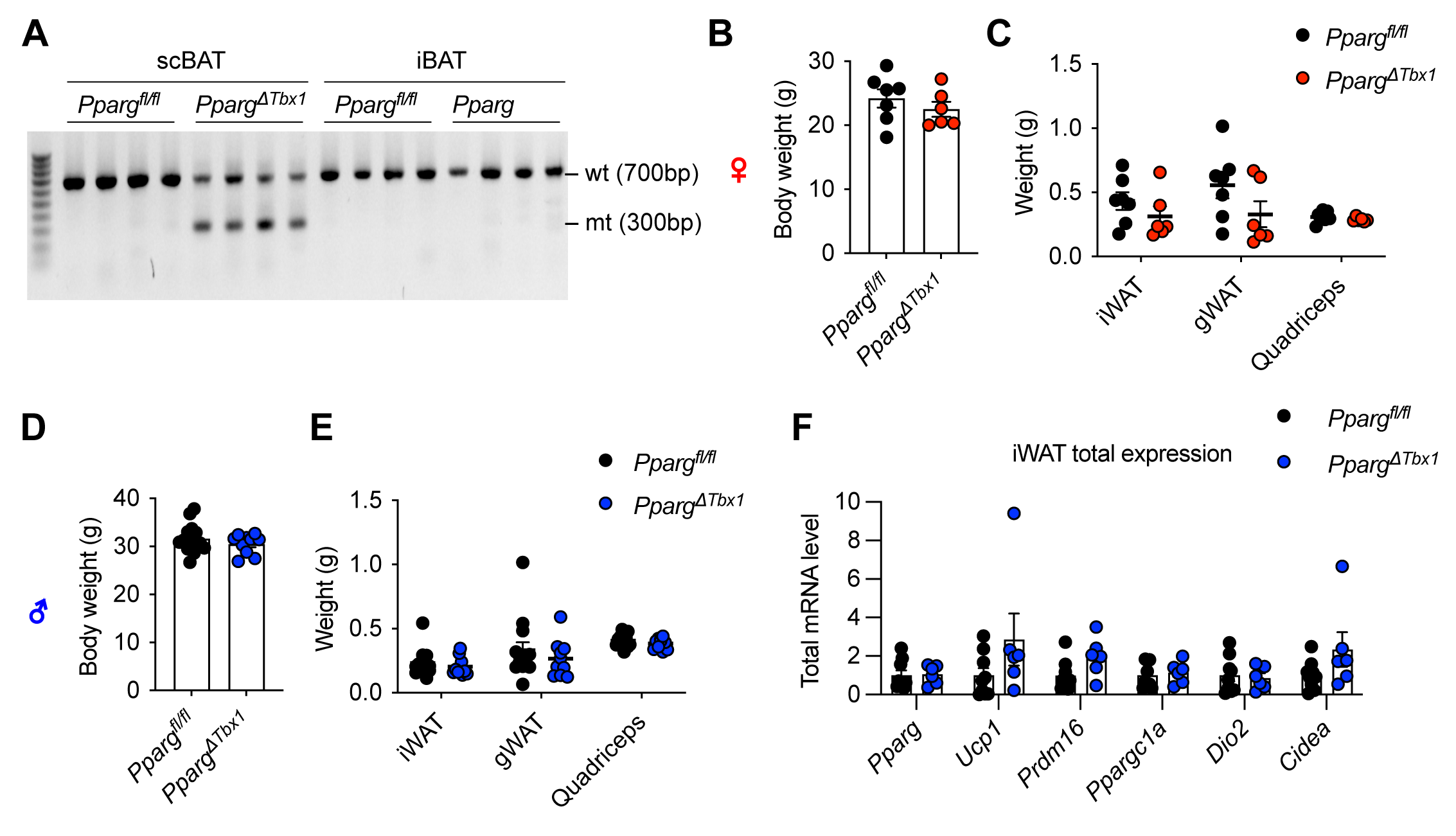

Supplement: S3 Fig — (A) Detection of wild type (wt, 700 bp) and mutant (mt, 300 bp) Pparg transcripts by RT-PCR. Note the approximately 50% recombination of the Pparg gene only in scBAT of PpargΔTbx1 mice. (B, C) Body weight (B) and tissue weight (C) of 4-month-old Ppargf/f (n = 7) and PpargΔTbx1 (n = 6) female mice. (D, E) Body weight (D) and tissue weight (E) of 4-month-old Ppargf/f (n = 14) and PpargΔTbx1 (n = 10) male mice. (F) Thermogenic gene expression in inguinal WAT of male mice was determined by RT-qPCR and adjusted by total tissue RNA to calculate the relative total transcript levels. (TIF) [file pbio.3002413.s003.tif]
