# Supplementary material for: Supraclavicular brown adipocytes originate from Tbx1+ myoprogenitors
Source: PLoS Biol. 2023 Dec 4;21(12):e3002413. doi: 10.1371/journal.pbio.3002413 (PMC10721186; doi:10.1371/journal.pbio.3002413)
Supplement: S1 Raw — (PDF) [file pbio.3002413.s005.pdf]

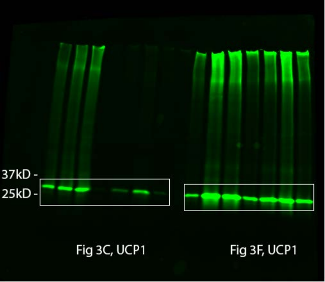
37kD -
25kD -
Fig 3C, UCP1
Fig 3F, UCP1

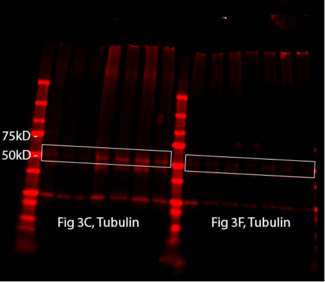
75kD -
50kD -
Fig 3C, Tubulin
Fig 3F, Tubulin

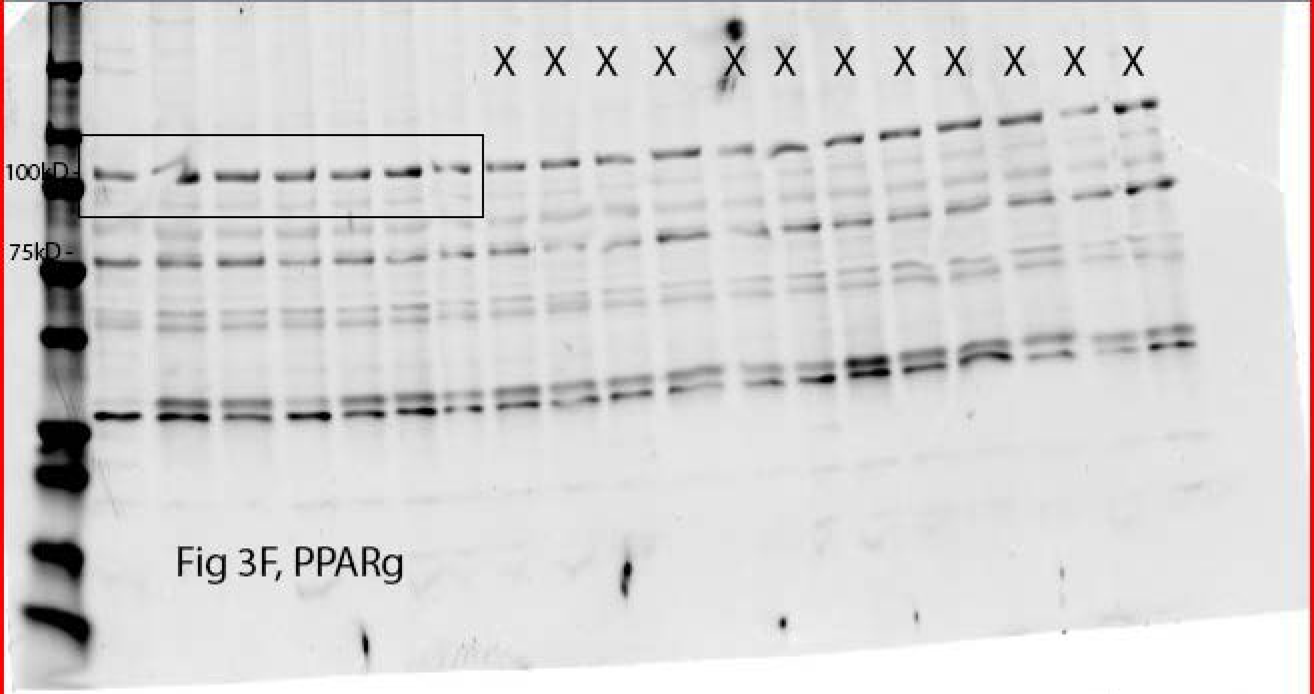
X X X X X X X X X X X X
100kD -
75kD -
Fig 3F, PPARg

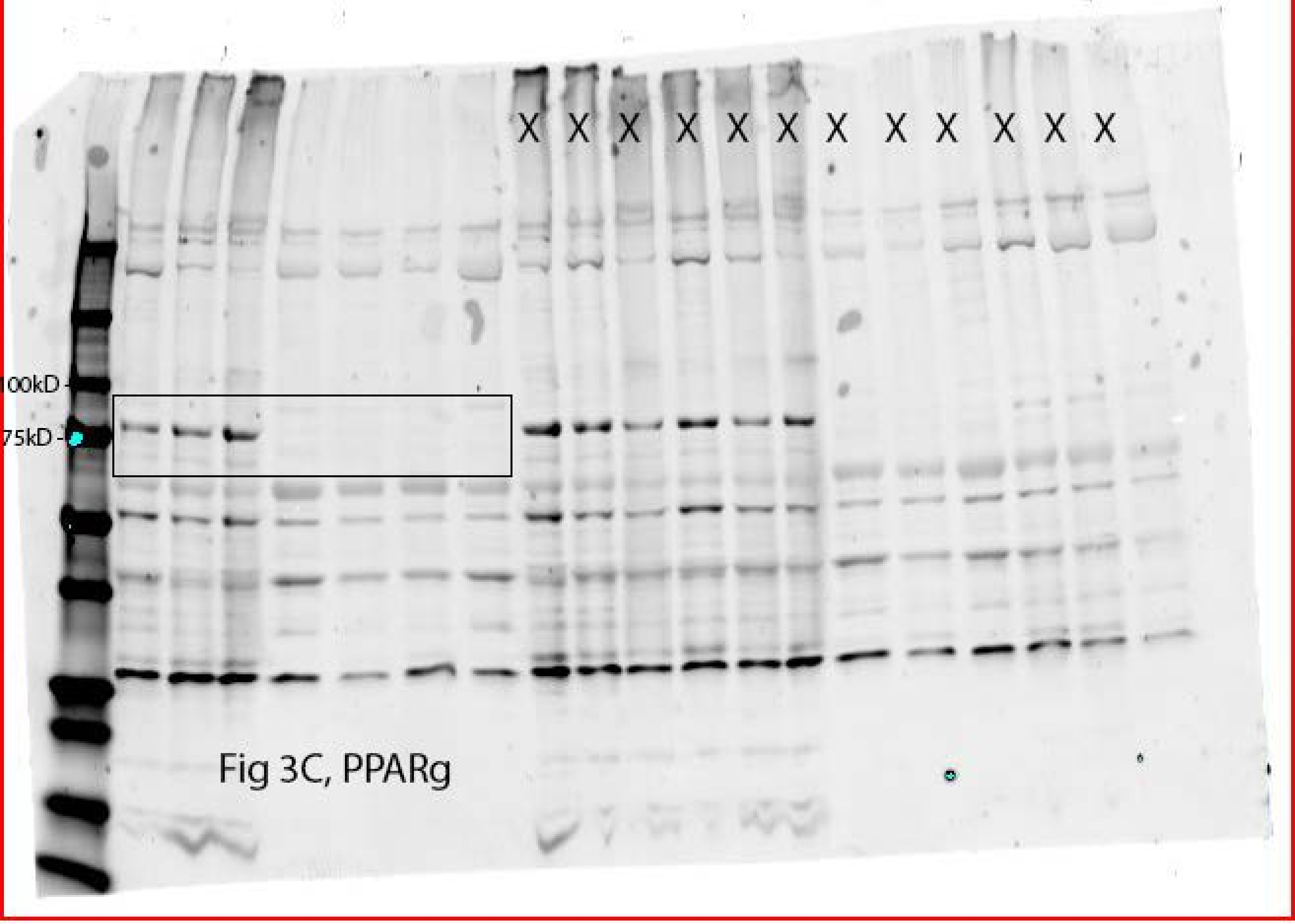
X X X X X X X X X X X X
100kD -
75kD -
Fig 3C, PPARg

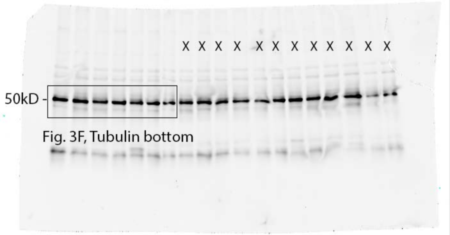
X X X X X X X X X X X X
50kD -
Fig. 3F, Tubulin bottom

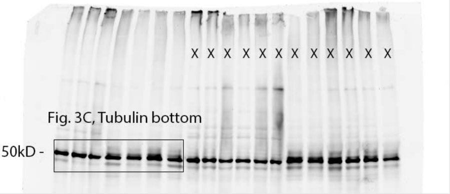
X X X X X X X X X X X X
Fig. 3C, Tubulin bottom
50kD -

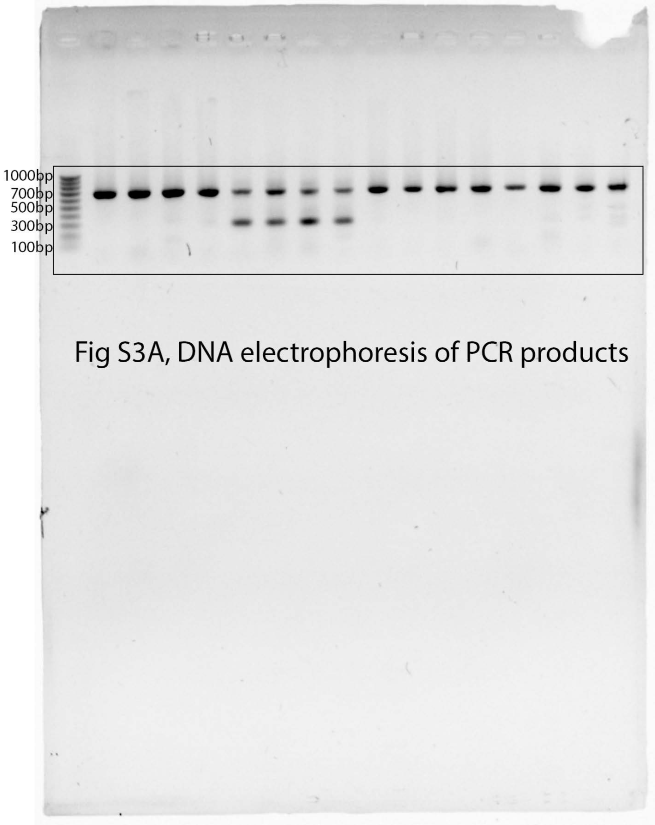

Fig S3A, DNA electrophoresis of PCR products
