# Supplementary material for: Supraclavicular brown adipocytes originate from Tbx1+ myoprogenitors
Source: PLoS Biol. 2023 Dec 4;21(12):e3002413. doi: 10.1371/journal.pbio.3002413 (PMC10721186; doi:10.1371/journal.pbio.3002413)
Supplement: Attachment — Submitted filename: 137483_1_rebuttal_2773041_rzhy9h.docx [file pbio.3002413.s006.docx]

**Responses to Reviewers’ Comments**

**Reviewer #1 (Evidence, reproducibility and clarity (Required)):**

*Summary
In this study, using genetic labeling and deletion modeling, the authors discovered that Tbx1 myoprogenitors, rather than Pax3+/Myf5+ cells, give rise to supraclavicular brown adipose tissue (scBAT). This finding is both intriguing and significant. Furthermore, the genetic ablation of PPARγ or PRDM16, driven by Tbx1-Cre, reduced the size/weight of scBAT and its thermogenesis function capacity, supporting the importance of Tbx-1 cells. Interestingly, the authors found that human scBAT, located in the deep neck region, exhibits higher Tbx1 expression than subcutaneous neck WAT, potentially indicating a similar origin of scBAT in rodents and humans. Overall, this novel finding is exciting and could push the BAT field into a new phase. The manuscript is also well-written and organized.

Comments
1. The authors assert that Myf5+ progenitors do not contribute to scBAT adipocytes. However, Figure 2C shows that 7% of medial scBAT are mG positive, suggesting a minor contribution of Myf5+ progenitors to scBAT. The conclusion that scBAT does not originate from Myf5+ precursor cells may be overly strong.*

**Response:** We thank the reviewer for pointing out the overstatement. We have rephased the conclusion from Figure 2 as “*Myf5*^+^ progenitors seldom give rise to scBAT adipocytes”.

*2. The authors claim that "prdm16 is dispensable for the development of scBAT." However, supporting data seems insufficient. The absence of a difference in scBAT weight does not guarantee that development was unaffected. Additional experiments, like H&E staining and immunofluorescence (IF) staining of RFP/GFP, could help demonstrate a similar number of GFP+ brown adipocytes in scBAT, thereby supporting this statement.*

**Response:** We do not have direct evidence that Prdm16 is dispensable for scBAT development; therefore, we have removed such statement in the revised manuscript. In addition, we provided H&E staining of scBAT, demonstrating evident adipose whitening and fibrosis in *Prdm16^ΔTbx1^* mice (**Figure 7C**). These results are consistent with previous findings that PRDM16 is required for the maintenance of brown adipocyte identity (PMID: 24703692).

*3. In the "scBAT contributes to temperature maintenance in mice" section, this phenomenon only seems to apply to female mice (Figure 5). This conclusion may need adjustment to account for this sex difference. Moreover, for females, are there H&E staining results available for scBAT? For males, is there a change in Ucp1 expression? It could be beneficial to examine mRNA expression levels for additional thermogenic genes, such as Dio2, Prdm16, Cidea, and PGC-1a.*

**Response:** Following the reviewer’s suggestion, we rephased our conclusion from Figure 5 to "scBAT contributes to temperature maintenance in **female** mice". Also as requested, we provided the H&E staining of female scBAT (**Figure 5E**), showing a partial loss of brown adipose identity in *Pparg^ΔTbx1^* mice. For males, we performed RT-qPCR and calculated total gene expression based on tissue weight. While *Pparg*, *Prdm16* and *Dio2* total expression was downregulated in *Pparg*-deficient scBAT, total *Ucp1* expression was unchanged in scBAT and iBAT of male mice (**Figure 5N, O**). This might explain the similar cold tolerance between wildtype and *Pparg^ΔTbx1^* males (**Figure 5P**).

*4. Considering that mutant mice have smaller scBAT, how does this difference influence glucose homeostasis between WT and mutants?*

**Response:** We performed glucose tolerance test and found no difference in glucose homeostasis of HFD *Pparg^ΔTbx1^* mice (**Figure 6C**). This could be due to intact iBAT in these animals, which is a much larger glucose consumer than scBAT.

*5. In Figures 5G & M, it seems more accurate for the y-axis label to read "Body temperature change."*

**Response:** We changed the y-axis label. Thank you for the suggestions.

*6. There is inconsistency in scale bar labeling:
o Figure 3A vs 3D
o Figure 4B vs 4C
o Figure 5L, where scale bars are missing in the H&E staining images
o Figure S1, where the scale bar is in the middle of the image*

**Response:** In the revised manuscript, we have provided consistent scale bars in all figures, including all these pointed by the reviewer. Thank you.

*7. In Figure 3, the western blot data should be quantified, and the molecular weight (kDa) should be included to clarify the band's position.*

**Response:** We have now indicated molecular weights in **Figure 3C, F** and provided quantified UCP1 and PPARγ expression levels to the right of Western blots.

*8. The statement "RT-qPCR revealed much higher levels of TBX expression in total lysates from deep neck BAT (Figure 8A)" could be clarified by adding "than neck WAT" at the end.*

**Response:** Done. Thank you for your suggestion.

**Reviewer #1 (Significance (Required)):**
 *Significance
The major advancement of this study lies in the authors' novel finding of the embryonic lineage of brown adipocytes in the supraclavicular brown adipose tissue (scBAT) depot. They demonstrated that this lineage differs from those in the dorsal BAT depots by utilizing Myf5-Cre, Pax3-Cre, and TBX1-Cre reporter mouse models.*


**Reviewer #2 (Evidence, reproducibility and clarity (Required)):**

*This study aimed to investigate the developmental origin of brown adipose tissue (BAT) in the supraclavicular region and its implications for metabolic health. The authors have used genetic fate mapping in mice to trace the lineage of brown adipocytes in the supraclavicular region.
The findings revealed that supraclavicular brown adipocytes do not originate from the Pax3+/Myf5+ epaxial dermomyotome, which is responsible for the development of interscapular BAT. Instead, most supraclavicular brown adipocytes were marked by the Tbx1+ lineage, indicating that the pharyngeal mesoderm is involved in their development. This work provides the first evidence that scBAT adipocytes do not share the same embryonic origins as iBAT fat cells. By identifying the location-specific myogenic progenitors for supraclavicular BAT versus interscapular BAT, the researchers shed light on the distinct developmental origins of different BAT depots. Overall, these findings provide new insights into the developmental origin of supraclavicular BAT and highlight the need to consider the anatomical locations and developmental origins in studying BAT development and function.

Major comments:
The manuscript addresses an important yet understudied area and provides convincing results that support the key conclusions. The authors also engage in extensive discussion, speculating on the broader implications of the findings and outlining the limitations of their study.
The observation that the loss of Pparg in Tbx1-expressing cells leads to a reduction in supraclavicular BAT is intriguing. However, as the authors acknowledge, Tbx1 is expressed in inguinal white adipocytes as well. Therefore, it remains unclear how changes in Tbx1 in ingWAT in this model might affect BAT depots. The authors should provide further clarification on this matter and include additional discussion regarding the potential indirect effects and limitations of these models.*

**Response:** We thank the reviewer for their overall enthusiasm about our manuscript, in which we primarily focus on *Tbx1* lineage cells in BAT, the supraclavicular depot to be more specific. *Tbx1-Cre* mediated genetic deletion of the *Pparg* gene will inevitably perturb PPARγ function in other *Tbx1*-expressing tissues, such as scapular muscles and certain WAT depots. In a recent publication (PMID: 32240964), the *Tbx1* gene was shown to be expressed in mouse inguinal WAT (iWAT) at a much higher level than interscapular BAT. TBX1 overexpression was sufficient to induce UCP1 protein in iWAT, while TBX1 deficiency rendered mice more sensitive to cold-induced body temperature drop. However, in our *Pparg^ΔTbx1^* mice, we did not observe any significant loss of *Pparg* gene expression in iWAT (**Figure S3F**), suggesting the possibility that *Tbx1*-positive cells are only a small proportion of iWAT. Nevertheless, we did not see any reduced expression of thermogenic genes in iWAT of *Pparg^ΔTbx1^* mice (**Figure S3F**), indicating either that *Tbx1*-expressing cells are dispensable for thermogenesis or that other still-undefined mechanisms exist to compensate for the loss of PPARγ. We acknowledge the limitations of our animal models and have discussed these points in the second paragraph of Discussion.

*In Figure 5, it would be beneficial to include the expression of a broader range of thermogenic genes and proteins in both males and females. This would strengthen the argument that the loss of Pparg in Tbx1+ progenitors impairs scBAT function.*

**Response:** Thank you for the suggestion and we have now provided the expression of additional thermogenic genes including *Ucp1*, *Prdm16*, *Ppargc1a*, *Cidea*, and *Dio2* in both males and females (**Figure 5F, G, N, and O**). These results demonstrate that loss of PPARγ in Tbx1+ myoprogenitors impairs scBAT thermogenic function, with more profound impact on males than females.

*Minor Comments:
It would be more appropriate to present the results of the cold challenge studies as absolute body temperature rather than just the difference between groups. This is important because there could be baseline differences in basal body temperature among the experimental groups.*

**Response:** Thank you for the suggestion. The results summarized in Figure 5H and 5P were from several batches of independent experiments that were carried out at different times. Plotting absolute body temperature created a bigger variance that precluded us from drawing any conclusion. Therefore, we decided to plot the data as changes in body temperature in Figure 5H and 5P. As shown in Figure 8, we did not observe baseline differences in body temperature between wildtype and *Prdm16^ΔTbx1^* mice, suggesting that loss of scBAT thermogenic function would not affect basal body temperature.

*It is recommended to include representative images of cre-negative animals to validate the reagents and models used in the study. Including such images would enhance the reliability of the experimental approach and strengthen the overall validity of the findings.*

**Response:** Representative images of Cre-negative mT/mG reporter animals have now been provided in **Figure S1**. Thank you for the suggestion.

*The manuscript would benefit from placing the findings in the context of the broader field. For instance, discussing whether the loss of Prdm16 in Myf5 progenitors has a similar impact on interscapular BAT (iBAT) development or thermogenic function would provide valuable insights. Additionally, exploring the relationship between Tbx1 progenitors and adipocyte progenitors in adult BAT depots, such as Pdgfra+ and Trpv1+ progenitors, could further enhance our understanding of the developmental origins and functional characteristics of different adipocyte populations*

**Response:** The reviewer raised an important point that we should place our finders in the context of the broader field. Indeed, it has been reported earlier that loss of PRDM16 in *Myf5* progenitors leads to iBAT dysfunction in adult mice (PMID: 24703692), a similar phenotype to what we found in scBAT of *Prdm16^ΔTbx1^* mice. The early embryonic development was not evidently perturbed in iBAT (using *Myf5-cre*) or scBAT (using *Tbx1-cre*), which might be compensated by other PRDM family protein, such as PRDM3. However, using *Adipoq-Cre* to knock out PRDM16 only leads to defects in beige adipocyte, but not classic BAT (PMID: 24439384). Together, these results demonstrate that PRDM16 in myoprogenitors is required for brown adipocyte identity maintenance during aging and similar transcriptional regulatory circuits control the differentiation and/or activity of both iBAT and scBAT. Further investigations are warranted to identify depot-specific regulations and functions of BAT. In adult BAT, progenitors that are marked by genes like *Pdgfra* and *Trpv1* have been reported to contribute to cold-induced BAT recruitment and tissue homeostasis. While not the scope of our current research, future endeavors are needed to test if embryonic *Tbx1*+ myoprogenitors give rise to all or only some populations of adult BAT progenitors. These points have now been added to the revised Discussion section.

**Reviewer #2 (Significance (Required)):**
 *Field of expertise: Adipose biology, Developmental biology, Thermogenesis

The manuscript addresses an important and previously understudied area in the field of brown adipose tissue (BAT) development. The study challenges the long-standing assumption that Pax3+/Myf5+ progenitors in the dermomyotome are the sole developmental source of brown adipocytes in mice, including both interscapular BAT (iBAT) and other brown adipocytes.

This work provides experimental evidence that the supraclavicular BAT (scBAT) adipocytes have a distinct embryonic origin compared to iBAT fat cells. While the developmental source of iBAT has been known for over a decade, this study demonstrates for the first time that scBAT adipocytes do not arise from the Pax3+/Myf5+ progenitors in the dermomyotome.

The findings presented in this manuscript have the potential to make a lasting impact on the brown adipose tissue research community, particularly those interested in the developmental aspects of brown fat.*


**Reviewer #3 (Evidence, reproducibility and clarity (Required)):**

*This manuscript explores the cell origin of supraclavicular brown adipose tissue (scBAT). UCP1-expressing brown/beige adipocytes are found in several anatomical locations but most studies have been focused on intrascapular brown adipose tissue (iBAT) in rodents, a depot that only exist in infants (but not adults) in humans. Very little is known about the biology (including the origin) of other BAT deposes. Here using multiple lineage tracing tools and conditional KO mice the authors convincingly demonstrate that about 50% of the scBAT cells originate from Tbx1+ myoprogenitors and provide functional evidence that ablation of Pparg or Prdm16 in Tbx1-cells blocks development of scBAT or affects thermogenic function of mice. The study design is straightforward, well carried out and the conclusion is supported by the data. The discovery has significant implication as brown adipocytes are mainly found in the supraclavicular region in the human body. I have few relative minor comments.

Tbx-Cre lineage tracing indicates that 50% scBAT cells are marked by Tbx1. The authors discussed this may be either due to low recombination efficiency of Tbx1-Cre or due to multiple lineage origin of scBAT cells. The possibility of Cre recombination efficiency can be addressed easily by genomic DNA recombination analysis.*

**Response:** To test recombination efficiency, we performed RT-PCR to detect wildtype and mutant *Pparg* mRNA using primers provided in the original report of the *Pparg*-floxed mice (PMID: 14660788). The mutant *Pparg* mRNA could only be detected in scBAT, not iBAT, of *Pparg^ΔTbx1^* mice (**Figure S3A**). The similar abundance of wildtype and mutant *Pparg* transcripts in *Pparg^ΔTbx1^* scBAT indicates a 50% recombination rate. However, we currently cannot distinguish the following possible reasons for the incomplete recombination: 1) only half of scBAT adipocytes are *Tbx1*-lineage cells; 2) a portion of *Tbx1*-progeny cells have low Cre expression and thus no recombination; 3) The *Tbx1* promoter does not fully recapitulate the endogenous *Tbx1* gene expression. The identity of non-*Tbx1* progeny adipocytes in the scBAT depot requires future investigations.

*In the introduction the authors conclude that Myf5, Pax3 are "location markers" instead of cell identity markers. While I agree with this statement it is quite confusing. It implies that the study aims to identify cell identify markers but based on the analogy Tbx1 is also a location marker.*

**Response:** We apologize for the confusion caused by using “identity” to describe both lineage origins and functional cell types. Based on previous and our current studies, we propose *Myf5*, *Pax3*, and *Tbx1* as location markers that distinguish BAT depots at different locations. They are not identity markers as these progenitors also give rise to other cell types, such as muscles, white adipocytes, fibroblasts, and others. We removed any statements indicating *Tbx1* as an identity marker.

*Figure 8 human expression compares TBX1 mRNA levels in neck fat to subcutaneous white fat. This is different from the comparison of dorsal and ventral BAT in mice. Therefore, the data are not particularly relevant to this study and should be moved to a supplemental figure.*

**Response:** We agree with the reviewer that the comparison of human *TBX1* was not made between dorsal and ventral BAT. This is due the loss of dorsal BAT in adult humans, which make it almost impossible to make such direct comparison in humans. Nonetheless, the higher expression of *TBX1* in deep neck BAT compared to subcutaneous neck WAT suggests that *TBX1* might be functionally important for BAT thermogenesis. This is consistent with a recent report using transgenic *Tbx1* mouse models that demonstrates the role of adipose TBX1 in thermogenic capacity (PMID: 32240964). We believe that data in Figure 8 is relevant to our current study, but we are also receptive to move it to the supplementary if the reviewer insists so.

*Can the authors speculate/discuss on why there is no Pax3 labelled cells but 7% Myf5 labeled cells in scBAT?*

**Response:** We would like to clarify that both *Pax3*+ (6.7%) and *Myf5*+ (7.2%) progenitors label a similar percentage of brown adipocytes in the medial scBAT. These percentages are now provided in the revised text and can be visualized in Figure 1H and 2D. On the other hand, nearly none of lateral scBAT adipocytes were labelled by either *Pax3*+ or *Myf5*+ progenitors.

**Reviewer #3 (Significance (Required)):**
 *The discovery has significant implication as brown adipocytes are mainly found in the supraclavicular region in the human body.*
